# Editorial for Special Issue “Effects of Probiotics on Health”

**DOI:** 10.3390/microorganisms12030442

**Published:** 2024-02-22

**Authors:** Piotr Heczko

**Affiliations:** Chair of Microbiology, Department of Bacteriology, Microbial Ecology and Parasitology, Jagiellonian University Medical College, 31-121 Kraków, Poland; mbheczko@cyf-kr.edu; Tel.: +48-12-633-25-67

Since 1987, when Professor Sherwood Gorbach discovered, characterized, and commercialized the first probiotic *Lactobacillus rhamnosus GG*, a total of over 17,000 publications have been indexed in PubMed under “probiotic” and “health”, which is an extensive amount of research on the specific bacteria and yeasts defined as “live microorganisms that, when administered in adequate amounts, exert a health benefit on the host” [1]. The publications on probiotics cover a broad spectrum of research subjects, from the fundamental mechanisms of their activity to clinical trials and animal observations [2,3,4]. Every progression in “omics” has had an immediate reflection in probiotic research.

Research on the mechanisms of probiotic activity has progressed in recent years since the formal status of postbiotics was secured by the definition proposed by the International Scientific Association of Probiotics and Prebiotics [5]. Postbiotics share most of the probiotic activities exerted in human and animal hosts. However, they can offer broader applicability than probiotics in various areas of health protection, being very stable in diverse formulations. Postbiotics have been addressed in around 1000 scientific articles issued in the past two years [6].

The expansive scope of scientific inquiry on the impact of probiotics and postbiotics on human and animal health encompasses a spectrum that closely parallels the fundamental challenges of contemporary medicine and nutrition (their pioneering role in the human gut microbiome [7], their oral health regulation, their influence on different immunity forms, their protection against a variety of neoplasms, and their added efficiency for animal feeding). The discovery of the gut–brain [8] and gut–skin [9] axes indicates an essential role for probiotics in these signaling mechanisms of the human body.

*Microorganisms* is one of the leading journals in which probiotics and their human and animal health properties have been addressed and grouped into Special Issues over several decades. This Special Issue, “Effects of Probiotics on Health”, includes 17 papers on pro- and postbiotics. They present current research on microorganisms and their products, different in vitro and in silico study approaches, animal experiments, and controlled clinical trials. In addition, several reviews and systemic reviews are published within the Issue. Some of the work detailed in this Issue is listed below:

- *L. rhamnosus’* postbiotic activity on fungal strains decomposing paper in historical manuscripts;
- Experiments showing the effects of *Prevotella histicola* on bone loss in mice with osteoporosis;
- *Roseburia intestinalis* and its postbiotic’s modulation of peptide YY expression, which stimulates satiation, using a sophisticated in vitro model of the human gut epithelium composed of four types of human cells;
- An in vitro demonstration that *B. breve* postbiotic protects against oxidative stress and lipid-associated proteins in a model of human neuroblastoma cells via PLIN4 gene regulation;
- A systemic review of the use of probiotics to prevent cognitive dysfunction in neurodegenerative diseases, addressing a significant symptom of these diseases.

This year, we commemorate 37 years of continuous advancement in exploring probiotics and their important role in maintaining health in host organisms. Hence, there is an opportunity to consider future research trajectories in this domain. An essential query arises regarding the specific proportion of microbial communities within the gut microbiota that possess tangible probiotic activities. Given the multitude of probiotic strains in the microbiome, investigating how their properties complement each other to orchestrate a concerted activity in safeguarding host health becomes a compelling area of interest. Clearly, the unresolved inquiries surrounding probiotics provide prospects for further exploration.
